# Structural, electrical and magnetic properties of Nd – A – CoO_3_ (A = Sr, Ca) Perovskite Powders by Mechanical Alloying

**DOI:** 10.1038/s41598-018-31458-7

**Published:** 2018-08-30

**Authors:** Celal Kursun, Musa Gogebakan, Esra Uludag, Mehmet S. Bozgeyik, Fatih Samil Uludag

**Affiliations:** 1 Department of Physics, Faculty of Art and Sciences, Kahramanmaras Sutcu Imam University, Kahramanmaras, 46100 Turkey; 2 Department of Electronics and Automation, Vocational School of Technical Sciences, Kahramanmaras Sutcu Imam University, Kahramanmaras, 46100 Turkey; 3 Department of Health Physics, Institute of Medical Sciences, Yildirim Beyazit University, Ankara, Turkey

## Abstract

In this work, NdCoO_3_ (NCO), Nd_0.8_Sr_0.2_CoO_3_ (NSCO) and Nd_0.9_Ca_0.1_CoO_3_ (NCCO) perovskite cobaltites were synthesised by mechanical alloying method. Structural evolutions, magnetic and electrical properties of these perovskite were systematically examined through X-ray diffraction (XRD), scanning electron microscopy with energy-dispersive X-ray detection (SEM-EDX), transmission electron microscopy (TEM), differential scanning calorimetry (DSC), vibration sample magnetometer (VSM) and impedance analyser (IA). The XRD and SEM results revealed that the microstructure of the perovskite materials changed during mechanical alloying. The average crystallite size of the perovskite materials was calculated by Debye Scherrer equation and was confirmed by TEM, and it was determined ~19 nm. From the VSM results, the all perovskites had soft ferromagnetic properties. IA measurements showed that relatively dielectric constants of the perovskites decreased with increasing frequency. Therefore, for the first time, nanostructured NCO, NSCO and NCCO perovskites exhibiting good properties were produced in only two steps which are milling and heating.

## Introduction

Rare earth perovskite cobaltite with the general composition of *Re*CoO3 and the doped mixed oxides with the general composition of *Re*_1−*x*_*A*_*x*_CoO_3_ have been extensively studied for their potential applications, where *Re* is for trivalent rare earth elements such as La, Pr, Nd, Eu, Gd, Ny and *A* is for divalent alkaline earth elements such as Ba, Sr, Ca, P or Mg. The potential applications of perovskite materials include cathodes in solid oxide fuel cells (SOFC), sensor materials for thermoelectric conversion, oxygen separation membranes, catalysts, non-volatile memories, magnetic memory media, refractory materials, automotive exhaust oxidation catalysts, oxygen electrode catalysts in aqueous alkaline solution batteries and solar cells^1–11^.

**Figure 1:**
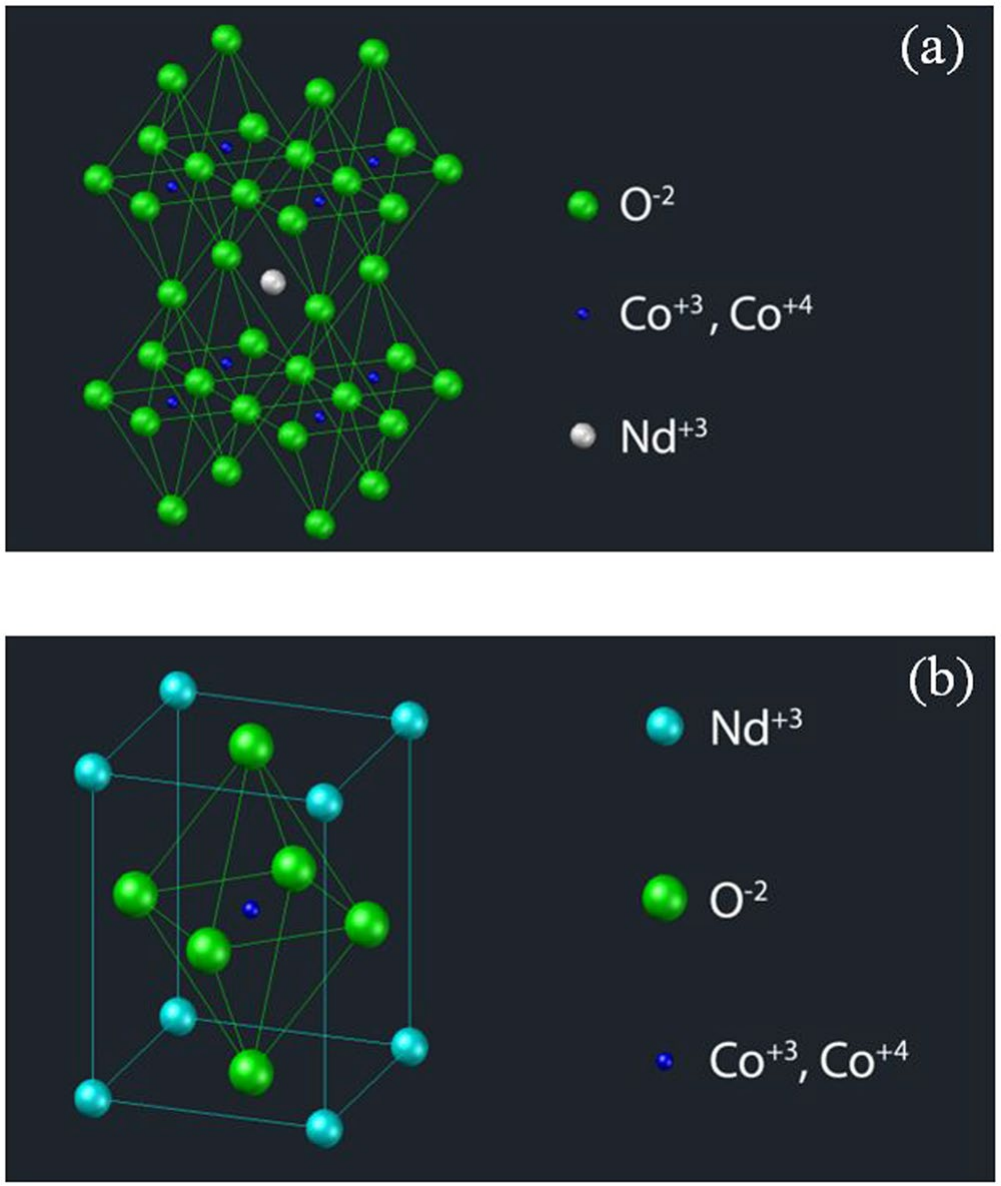
(**a**) Ideal cubic perovskite structure. (**b**) Ideal cubic structure in primitive cell.

The ideal perovskite structure is shown in Fig. 1a. The primitive cell of the ideal cubic perovskite structure can be seen in Fig. 1b. In ideal perovskite-type structure, A cation is 12-fold coordinated and B (Co) cation is 6-fold coordinated with oxygen anions. The perovskite oxides exhibit super conductivity, colossal magneto resistance (CMR), ferroelectric, photo catalytic and unique thermoelectric (TE) properties^12–15^. In addition, for some cobaltites like NdCoO_3,_ the high Seebeck coefficient was reported near the room temperature^16–18^. The ferromagnetic cobaltites have been drawing attention for their large magnetic moment and colossal magneto resistance effect (CMR). The cobalt oxides have properties of hopping of e_g_ electron double-exchange interactions (DE) between Co^+3^ and Co^+4^ ions which depend on the Co-O-Co bond angle and Co-O bond length like in manganates^18,19^.

Both trivalent and tetravalent cobaltites exhibit two magnetic transitions with increasing temperature. One of them is the spin-state transition, also known as the metal-insulator transition, which is a paramagnetic-ferromagnetic (PM-FM) phase transition (Jahn-Teller distortion) at the critical Curie temperature (T_C_). The second transition is about the low spin state, intermediate spin state and high spin state that exist in Co ions. However, the low spin state (LS) is t_2g_^6^ e_g_^0^ with S = 0 for Co^+3^ ion and t_2g_^5^ e_g_^0^ with S = 1 for Co^+4^ ion, the intermediate spin state (IS) is t_2g_^5^ e_g_^1^ with S = 1 for Co^+3^ ion and t_2g_^4^ e_g_^1^ with S = 3/2 for Co^+4^ ion and the finally, high spin state (HS) is t_2g_^4^ e_g_^2^ with S = 2 for Co^+3^ ion and t_2g_^3^ e_g_^2^ with S = 5/2 for Co^+4^ ion^20–25^. These are nearly degenerate in energy. When the crystal structure changes, the spin transitions can be observed. Because of this reason both the spin state and the ionic radius of Co^+3^ ion increase (Ionic radius for Co^+3^ r_LS_ = 0.545 Å, r_IS_ = 0.560 Å and r_HS_ = 0.610 Å). This change can be explained by the Hund energy in the CoO_6_ octahedron, Jahn Teller effect, double-exchange mechanism and collerated polarons^26–28^.

When the ferromagnetic transition temperature (T_C_) decreases, B-O-B (Co-O-Co) bond angle and hence Goldschmidt factor introduced a tolerance factor, “t” also decreases. However, the distortions in the lattice are determined owing to the value of tolerance factor. The tolerance factor is calculated by Eq. 1,

1 $$t=\frac{{r}_{A}-{r}_{0}}{\sqrt{2}({r}_{B}-{r}_{0})}$$

in consequence of the relation between the ionic radii holds:

2 $${r}_{A}+{r}_{0}=\sqrt{2}({r}_{B}+{r}_{0})$$

where *r*_*A*_ is the ionic radius of A site cation (A = Nd, Sr, Ca), *r*_0_ is the ionic radius of oxygen and *r*_*B*_ is the ionic radius of B site cation (B = Co).

**Figure 2:**
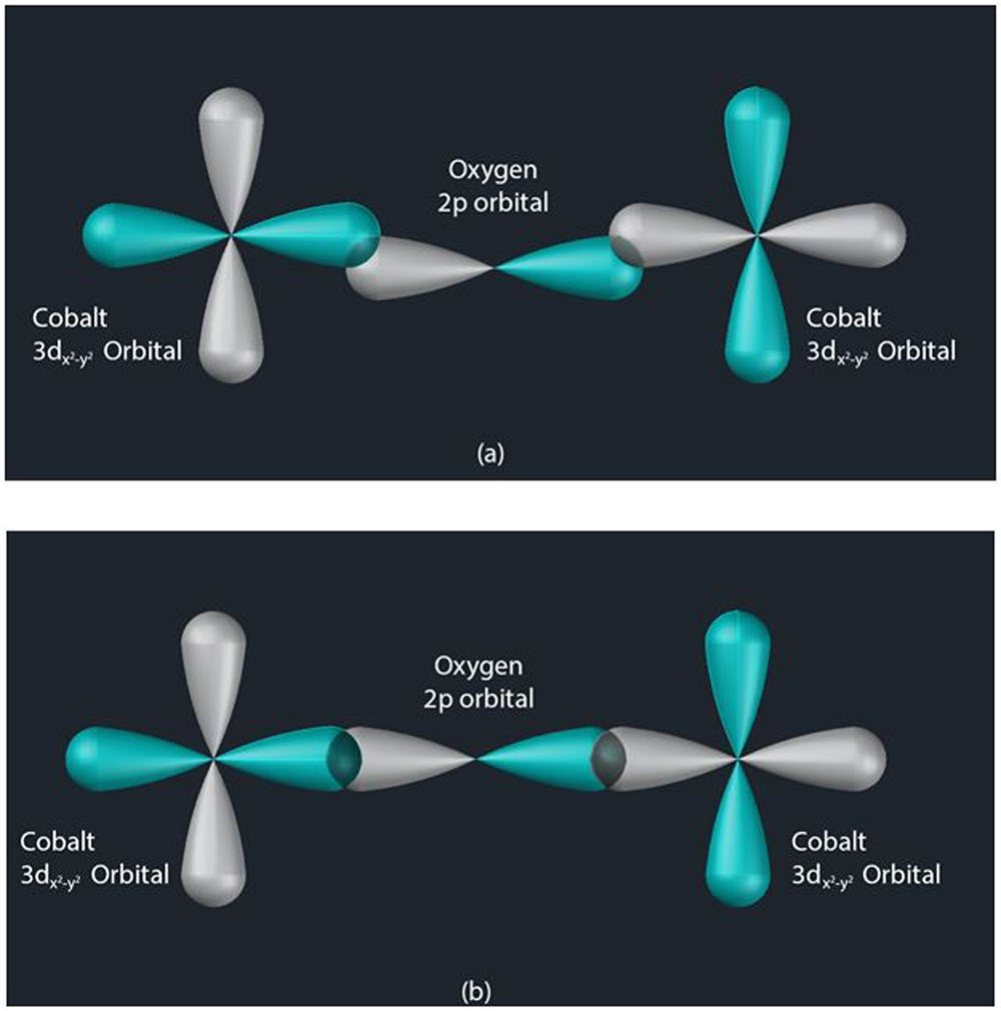
The overlap influence of Co-O-Co angle. (**a**) When the Co-O-Co angle can not achieved 180°, overlap is small. (**b**) While the Co-O-Co angle is 180°, overlap is well.

The value of tolerance factor “t” is stable between 0.75 and 1.0. If the value of tolerance factor is 1, the bond angle is 180°. When the angle is 180° or approaches to this value, the best overlap is achieved^29–32^. The effect of the value of the tolerance factor on bond angles were schematized in Fig. 2. It can be obviously seen in Fig. 2 that the bond angle is not 180° (Fig. 2a) if the value of tolerance factor doesn’t close to 1, however, if it is 1 (Fig. 2b), the best overlap exists.

The perovskite oxides can be synthesised by following methods: solid state reactions, a citric acid assisted soft chemistry synthesis, the wet chemistry-polymerizable complex method, the glycine- nitrate process method, citrate- nitrate combustion method, reactive grinding, the polymeric precursor method, sol-gel method, electrospinning method, polymerized gel combustion, co-precipitation, glycothermal etc.^33–44^. However, in the present study, mechanical alloying (MA) method was used to produce these materials as a new technique.

**Figure 3:**
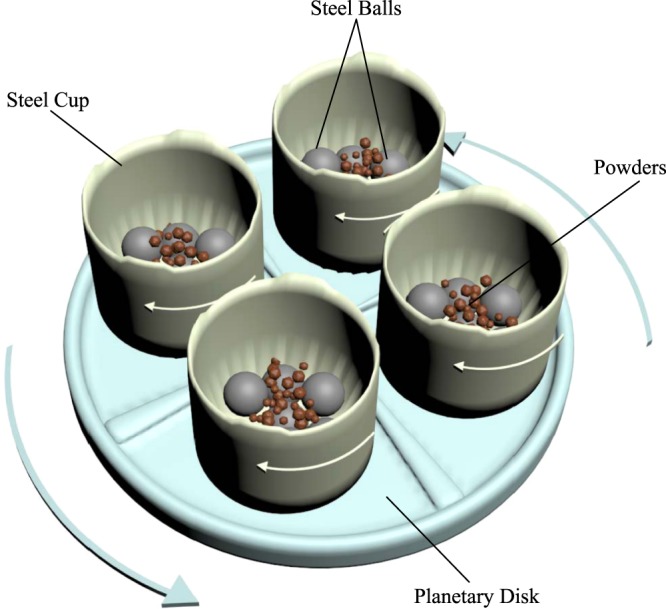
Schematic diagram of mechanical alloying.

**Figure 4:**
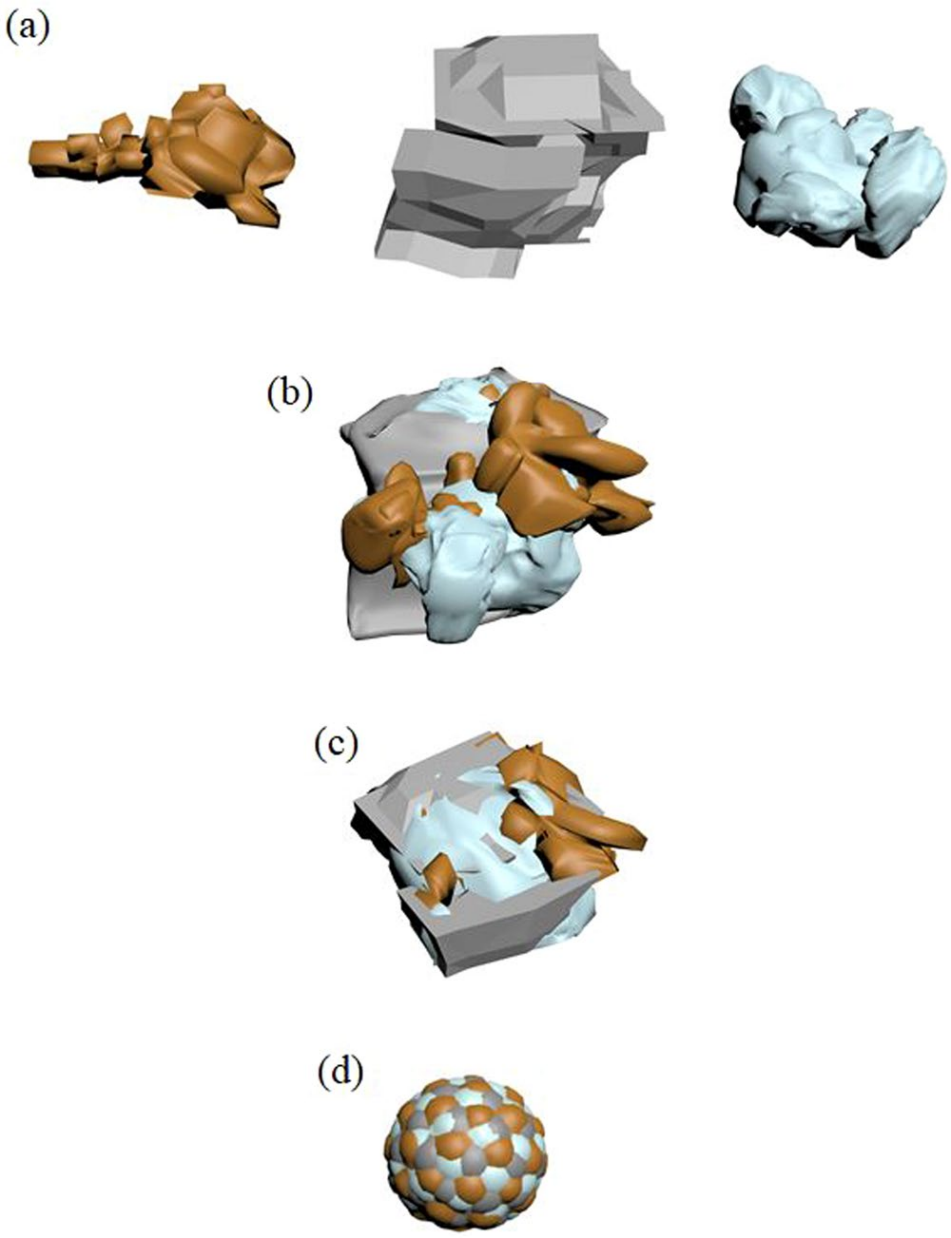
Schematic diagram of the microstructure evolution for powders during MA process.

MA has been used to synthesis quality powders of compounds and alloys with well-controlled microstructures and morphologies^45^. In this technique, materials are produced on powder forms, which can be easily compacted in desired shapes and dimensions for practical application. Working principle of MA is schematized in Fig. 3. As shown in Fig. 3, the powders in the stainless steel cups remain between balls. The balls collide with each other while the cups and planetary disk are turning opposite direction. Afterwards the powders are diffused into each other with the effect of colliding balls. The structural changes of the powders which subject to the ball collisions during the milling operation are schematized in Fig. 4. The powders have different size distribution and shapes at initially (Fig. 4a), however, their particle size and shapes change with increasing milling time and they diffuse into each other because of the cold welding and fracturing of the particles as it seen Fig. 4b and c. With further milling time it can be seen that the particle becomes smaller and it appears to approach a spherical shape in Fig. 4d. In this final stage, the homogeneity of the powders is the highest.

For the first time the perovskite cobaltites such as Nd – A – CoO_3_ (A = Sr, Ca) are produced in the form of powder by this method without any pre-annealing or sintering, acidic solution and alcohol for solvation. This technique allows to obtain the perovskites in only two steps: milling and heating. In addition to the easy production of the perovskite cobaltite oxides by mechanical alloying, the microstructure analysis can also be done easily^45,46^.

## Methods

The perovskite cobaltates, NdCoO_3_, Nd_0.8_Sr_0.2_CoO_3_ and Nd_0.9_Ca_0.1_CoO_3_ were prepared by mixing Nd(NO_3_)_3_.6H_2_O (Aldrich, 99.9%), Sr(NO_3_)_2_ (Acros Organics, 99.8%), Ca(NO_3_)_2_.4H_2_O (Acros Organics, 99.9%) and Co(NO_3_)_2_.6H_2_O (Merck, 99.7%) which are measured stoichiometric amount of nitrate salts. The mixture of the powders with the stainless steel balls was charged into a stainless steel cup (125 ml). The powders were mechanically milled in planetary ball mills (Fritsch Pulverisette 5) at room temperature. A ball to mass powder ratio is 5:1. This method was performed at the rotation speed of 300 rpm and the whole mechanical milling process continued for 4 h, 8 h, 12 h, 24 h and 48 h for each compounds. Moreover, after each 20 min of ball milling, mechanical milling was interrupted for 20 min in order to cool down the vials. After production processes by ball milling, mechanically milled for 48 h NCO, NSCO and NCCO powders were heated with the heating rate of 20 °C min^−1^ up to 1000 °C, which the powders were annealed for 2 h in a furnace with argon atmosphere.

Crystalline phases of the whole samples during milling were identified by X-Ray diffraction (XRD) using a Philips X’Pert PRO diffractometer with Cu Kα radiation (λ = 0.154 nm). The thermal behaviour was examined by Perkin-Elmer Sapphire differential scanning calorimetry (DSC) under inert gas atmosphere using continuous heating mode with the heating rate of 40 K min^−1^ for all perovskite powders. The microstructural analysis was studied by SEM with a JEOL JCM 5000 scanning electron microscope at an acceleration voltage of 10 kV. Transmission electron microscopy (TEM) investigation of the NdCoO_3_ was performed by a Phillips CM-20 operating at 200 kV. For dielectric study samples were mixed with polyvinyl alcohol (PVA) as a binder and pressed into cylindrical pellets of 13 mm diameter, under an isostatic pressure of 5 × 10^6^ Nm^−2^ using a hydraulic press. Then the pellets were sintered at an optimized temperature of 400 °C for 30 min. in air. Dielectric properties were measured at room temperature in a wide frequency range of 100 Hz to 1 MHz using an impedance analyser HP4294A. Magnetic characteristics were studied up to a field of 1.5 T by Vibrating Sample Magnetometer (VSM) Lake Shore 7400.

## Results and Discussion

### Microstructural evaluation

**Figure 5:**
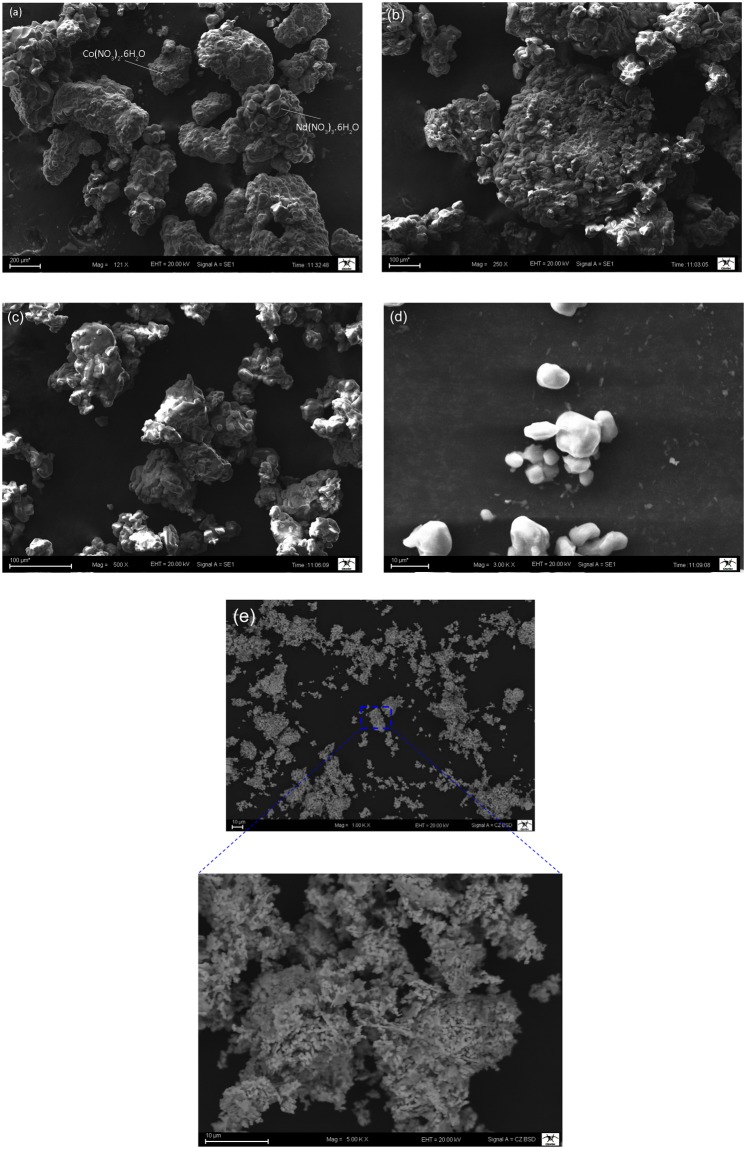
Morphological changes of the NdCoO_3_ (NCO) powders as a function of the milling time (**a**) 0 h; (**b**) 8 h; (**c**) 24 h; (**d**) 48 h; (**e**) after heat treatment at 1000 °C for 2 h for 48 h milling.

The microstructural evolutions of the NCO, NSCO and NCCO powders were performed by SEM to reveal the morphological changes and the decrease in the particle size during mechanical milling. Figure 5 presents the SEM micrographs of un-doped NdCoO_3_ powders. As shown in Fig. 5a, the unmilled Nd(NO_3_)_3_.6H_2_O and Co(NO_3_)_2_.6H_2_O salt particles had irregular morphology. However, Nd(NO_3_)_3_.6H_2_O powder particles were nearly uniform in size with hexagonal morphology. Besides, the sizes of Co(NO_3_)_2_.6H_2_O powders were smaller than the sizes of Nd(NO_3_)_3_.6H_2_O powders. After 8 h of milling (Fig. 5b), the particles begin to agglomerate because of the cold welding by colliding balls. Thus, the microstructure of powders was changed. The colliding force between balls and Nd(NO_3_)_3_.6H_2_O and Co(NO_3_)_2_.6H_2_O powders were largely used in the plastic deformation process. For the powders subjected to 24 h of mechanical milling, it can be seen clearly in Fig. 5c that the average particle size of the powder decreased due to fracturing. At this stage, the powder attached strongly each other and they had absolutely different shapes from initial form. For the higher milling time up to 48 h, the size distribution and shapes of the powders have continued to change. As it seen Fig. 5d after 48 h of milling it resulted in the formation of submicrometer particles with an average particle size of ~10 μm. One can obviously see that the powder particles had more homogeneity and appeared to a spherical shape in Fig. 5d. Figure 5e shows SEM images of perovskite NCO powders after heat treatment at 1000 °C for 2 h after 48 h milling. According to Fig. 5e, the spherical shape of the powders (in Fig. 5d) was changed and transformed to shapeless form with porous like a sponge rubber.

In the rare-earth perovskite with general formula ABO_3_, the structural distortions are usually occurred. While Sr or Ca replace Nd in the crystal lattices of Nd_0.8_Sr_0.2_CoO_3_ and Nd_0.9_Ca_0.1_CoO_3_, respectively, the average ionic radius in A site changes. Similarly, these ionic mismatches were observed for the doped perovskite cobaltites. The ionic radii mismatch in A-site lead to size variance σ^2^ which is

3 $${\sigma }^{2}=(\sum _{i}{x}_{i}{{r}_{i}}^{2})-{\langle {r}_{i}\rangle }^{2}$$

The ionic radius of the A site and B site elements and their coordinates are listed in Table 1.

**Table 1:** Ionic radius and coordinates of A site and B site elements.

Elements	Nd^+3^	Sr^+2^	Ca^+2^	Co^+3^	Co^+4^	O^−2^
Coordinate	IX	IX	IX	VI	VI	VI
Ionic Radius Angstrom (Å)	1,163	1,31	1,18	0,545	0,53	1,4

Table 2 shows the tolerance factors and ionic mismatches for each of the perovskite samples. It is well known that the tolerance factor changes between 0.75 and 1.00^47^. According to the Table 2 the tolerance factors of Nd – A – CoO_3_ (A = Sr, Ca) are in the range of 0.93–0.94. These results are consonant with literature.

**Table 2:** Tolerance factors and ionic mismatches of perovskite materials.

Content (Sr, Ca)	〈r_A_〉 (Å)	〈r_B_〉 (Å)	r_O_ (Å)	t (tolerance factor)	Ionic mismatch (σ^2^) Å^2^
0.0	1,163	0,5450	1,4	0,9318	0
0.2 Sr	1,1924	0,5420	1,4	0,9439	0,00345
0.1 Ca	1,1647	0,5435	1,4	0,9331	0,00002

**Figure 6:**
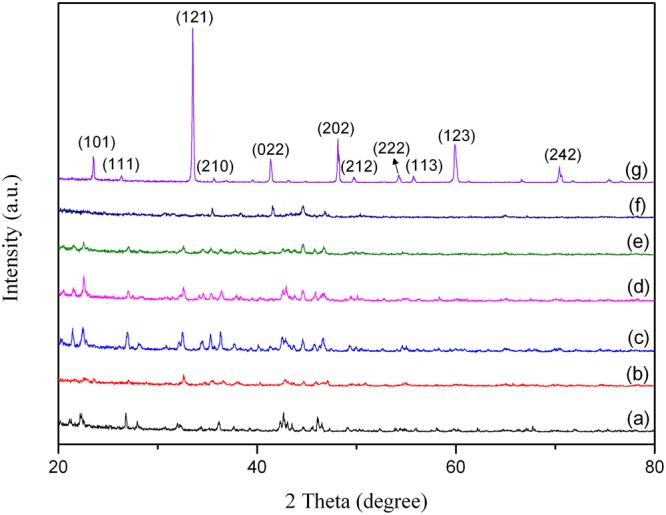
X-ray diffraction patterns of NdCoO_3_ (NCO) powders as a function of mechanical alloying time: (**a**) 0 h; (**b**) 4 h; (**c**) 8 h; (**d**) 12 h; (**e**) 24 h; (**f**) 48 h and (**g**) subsequent 2 h heat treatment at 1000 °C for 48 h milling.

**Figure 7:**
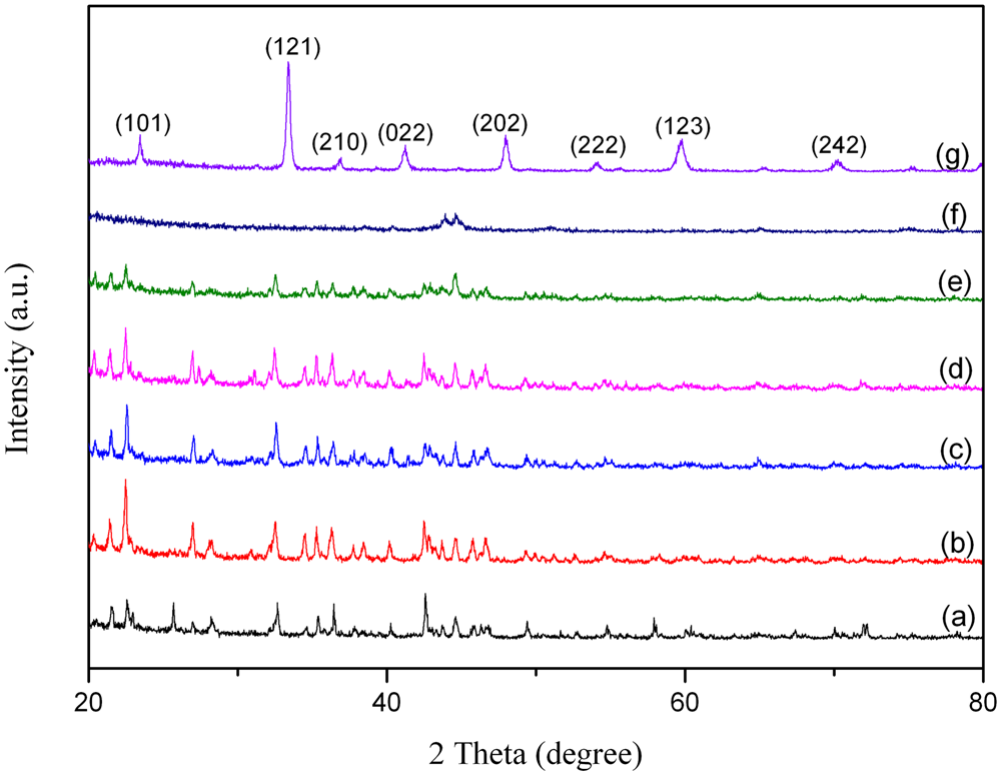
X-ray diffraction patterns of Nd_0.8_Sr_0.2_CoO_3_ (NSCO) powders as a function of Mechanical alloying time: (**a**) 0 h; (**b**) 4 h; (**c**) 8 h; (**d**) 12 h; (**e**) 24 h; (**f**) 48 h and (**g**) subsequent 2 h heat treatment at 1000 °C for 48 h milling.

**Figure 8:**
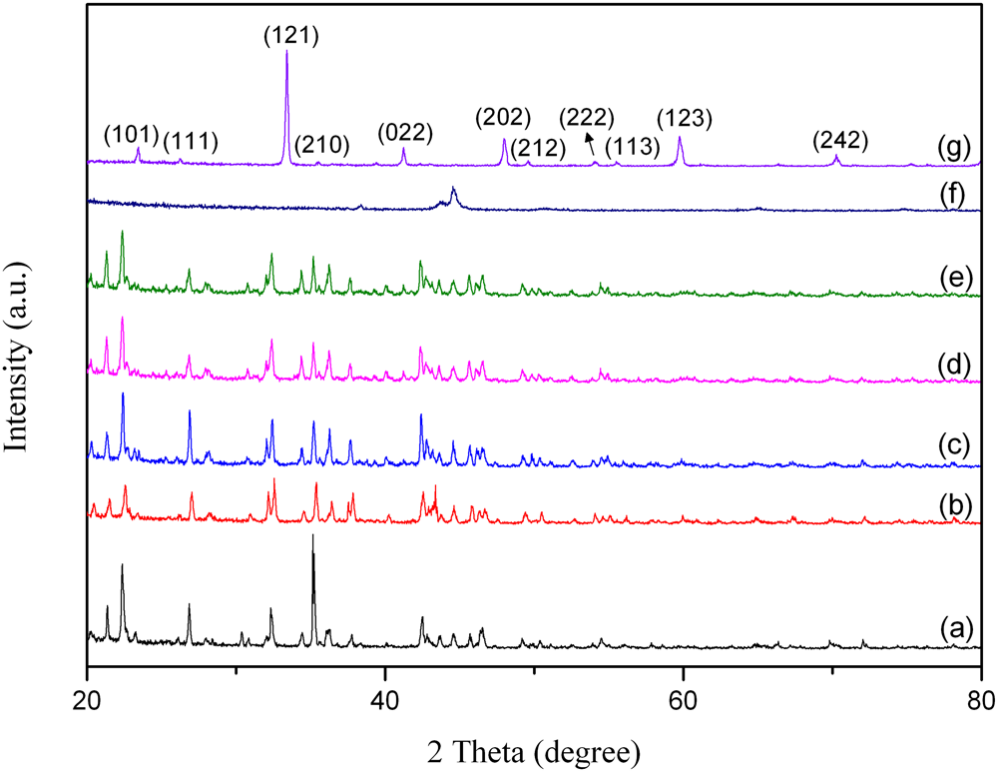
X-ray diffraction patterns of Nd_0.9_Ca_0.1_CoO_3_ (NCCO) powders as a function of Mechanical alloying time: (**a**) 0 h; (**b**) 4 h; (**c**) 8 h; (**d**) 12 h; (**e**) 24 h; (**f**) 48 h and (**g**) subsequent 2 h heat treatment at 1000 °C for 48 h milling.

The structural features of NCO, NSCO and NCCO perovskite powders were characterised by X-ray diffraction. Figures 6–8 show the XRD patterns of the NCO, NSCO and NCCO perovskites. As shown in Figs 6–8, all the mechanically milled the powders indicate certain behaviours during mechanical milling. Because of the heavy deformation, repeated fracturing and cold welding of the powders, the diffraction lines shift and the peak broadenings increase as well as the diffraction lines of solute disappear with increasing milling time. As a result of the increase in internal strain and reduction in grain size, the peaks broadening of the XRD peaks has taken place during mechanical alloying. The XRD patterns of 2 h heat treatment at 1000 °C after 48 h milling for perovskite NCO, NSCO and NCCO powders are also shown in Figs 6g, 7g and 8g, respectively. These perovskite peaks are in agreement with the earlier reported studies for NdCoO_3_ perovskite materials by salt-assisted combustion process^44^ and sol-gel technique^46^.

According to the results of 0 h–24 h milling of the powders, the sharp crystalline peaks which belong to nitrate salts broadened and their intensities decreased in Figs 6(a–e), 7(a–e) and 8(a–e). It can be seen clearly in Figs 6–8(f) that most of the elemental peaks disappeared after 48 h of milling. These peaks which disappear after 48 h milling time is usually attributed to the creation of nanostructured solid solution^48^. The all powders subjected to heat treatment at 1000 °C for 2 h after 48 h milling. From the XRD patterns of NCO, NSCO and NCCO powders after this process, it can be obviously seen in Figs 6–8(g) that the crystallite peaks with very sharp and high intensity were obtained. These peaks are characteristic peaks of the perovskite materials which are identified by ICSD code 082078^49^. The XRD analyses reveals the formation of orthorhombic NdCoO_3_ (space group: Pnma) in all samples (Figs 6–8(g)).

**Figure 9:**
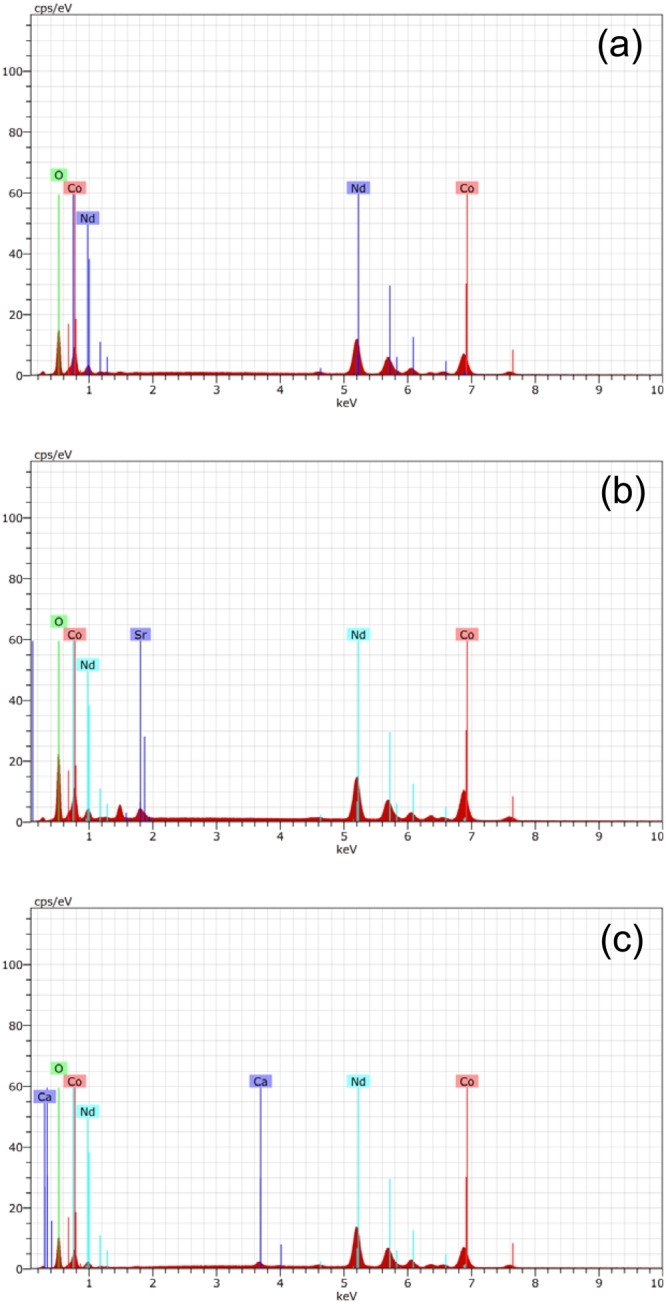
EDX analysis of the perovskite NCO, NSCO and NCCO powders.

Figure 9a–c shows the EDX analysis of the perovskite NCO, NSCO and NCCO powders. According to Fig. 9a–c, the elemental peaks which belong to Nd, Co and O can be seen clearly for all perovskite powders. However, Sr and Ca elemental peaks are also observed for NSCO and NCCO perovskites, respectively. It is because these elemental peaks exist in their composition in contrary NCO perovskite which are occured Nd, Co and O. The observed Sr and Ca elements from EDX analysis couldn’t be identified in XRD pattern (Figs 6–8) because of their very few amount in the NSCO and NCCO. It may be also attributed that Sr and Ca solve into the Nd or Co elements. The similar solving process was observed in previously work at mechanical alloying systems in literature^45,48^.

### Crystallite size evaluation

The crystalline sizes of NCO, NSCO and NCCO powders are calculated from broadening of XRD peaks by Scherrer equation^45^

4 $$D=\frac{0.9\lambda }{B\,\cos \,\theta }$$

where *D* is the average crystallite size, *λ* the wave length of using X-ray, *B* the full width (in radians) at half maximum intensity and *θ* the diffraction Bragg angle.

**Figure 10:**
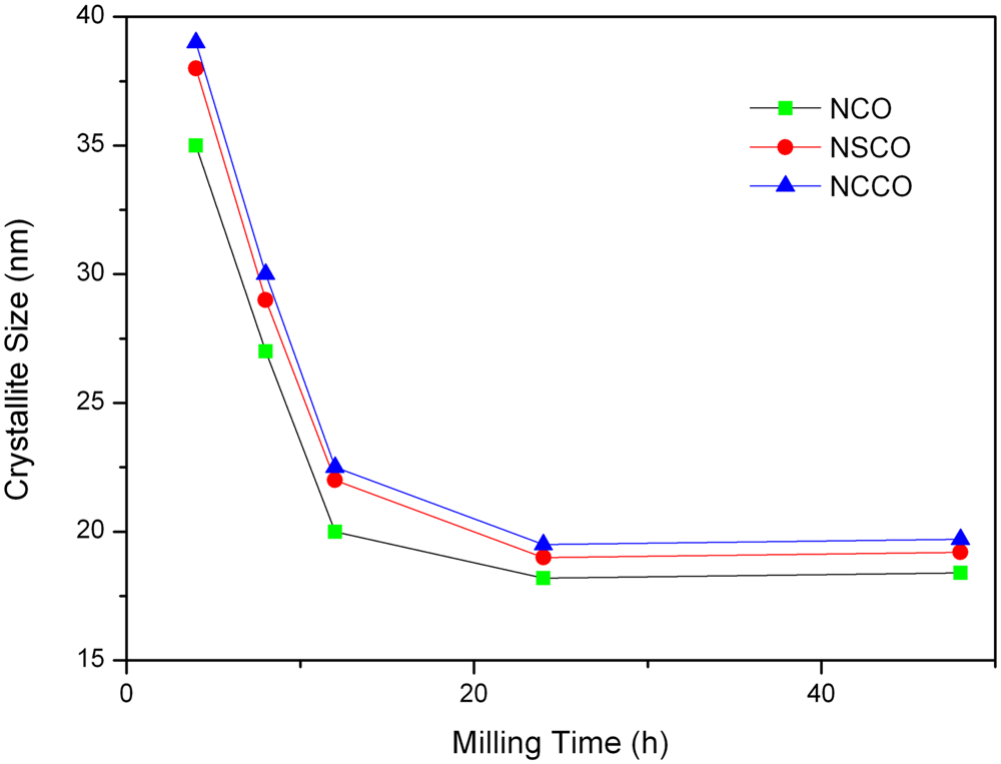
Crystal size of mechanically milled NCO, NSCO and NCCO powders as a function of milling time.

**Figure 11:**
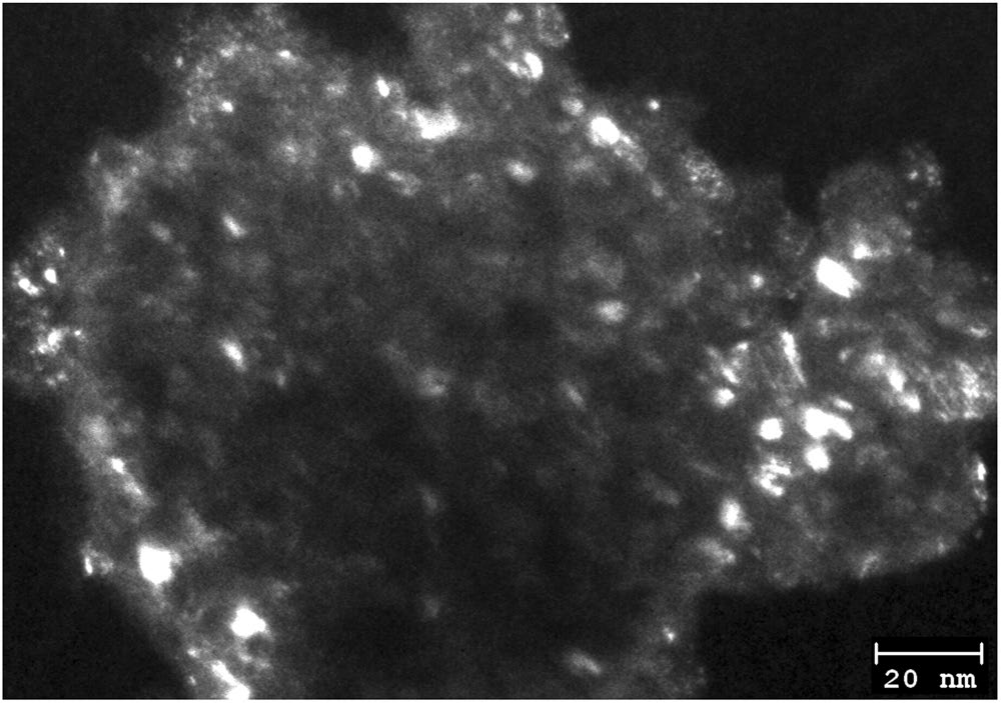
Typical dark field TEM image of the perovskite NCO for 48 h milling.

The crystallite size evolutions of NCO, NSCO and NCCO powders as function of milling time are presented in Fig. 7. As seen from Fig. 10, the crystallite sizes of the powders decreased sharply and were calculated ~20 nm after 12 h ball milling. The average crystallite sizes of NCO, NSCO and NCCO powders reach the lowest values, 18.2 nm, 19.2 nm and 19.7 nm, respectively, after 48 h of milling time. The crystallite size evolution of NCO powder was also monitored by TEM to predicate the crystallite size of obtained by XRD. Figure 11 shows typical dark field TEM image of the NCO for 48 h milling. As it seen clearly Fig. 11 the crystallite size of the NCO is below 20 nm. This result is consonant with the crystallite size value of NCO calculating by XRD data.

### Magnetic and electrical properties

**Figure 12:**
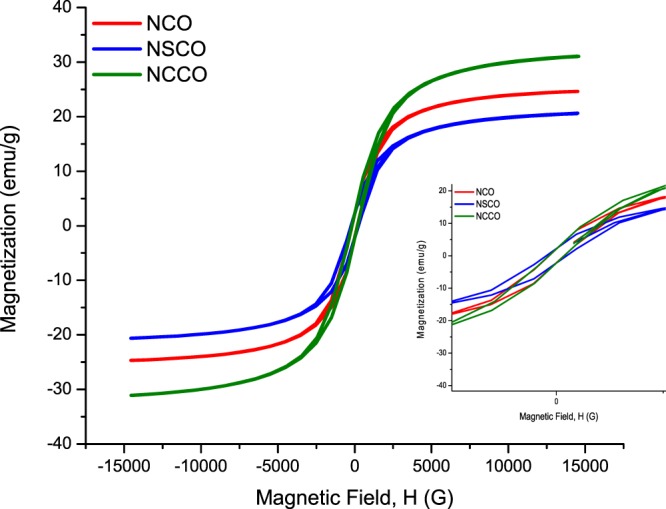
Room temperature magnetization vs. magnetic field (M-H) measurements of NCO, NSCO, and NCCO.

Figure 12 indicates steeply increasing magnetization in very narrow hysteresis loops. According to the related hysteresis loops in Fig. 12 and Table 3 low coercivity (HC) ranging from 170 to 244 G and small remnant magnetization (Mr) between 2.085 and 2.254 emu/g revealed that the magnetic behaviour looks like soft ferromagnetic.

**Table 3:** Tabulated hysteresis loop parameters.

Sample	H_C−_ (G)	H_C+_ (G)	M_r−_ (emu/g)	M_r+_ (emu/g)	M_S−_ (emu/g)	M_S+_ (emu/g)	M_r-_/M_S−_	M_r+_/M_S+_
NCO	−170	178	−2.143	2.085	−24.631	24.586	0.087	0.085
NSCO	−233	244	−2.249	2.196	−20.551	20.649	0.109	0.106
NCCO	−177	184	−2.254	2.148	−31.017	31.124	0.073	0.069

These values are far away from those related values of superparamagnetics which have theoretically no hysteresis loop by means of zero remanence and zero coercivity, i.e., just a sigmoidal S like shape. As a matter of fact, anisotropy, interaction of identical particle, and crystallite size distributions have the potential to alter the M-H hysteresis curves. Hence, experimentally negligible small (almost zero) remanence magnetization and coercive field (a few Oersted) have been measured for ferro/ferrimagnetic nanoparticles which have the size distribution ranging from a few nanometers to few tenth of nanometers. Such a nanoparticle system behaves superparamagnetic hysteresis curve. Moreover, soft ferromagnetic materials composed of nanoparticles below certain size could show superparamagnetic behaviour.

On the contrary, according to Fig. 12 and Table 3 all samples present appreciable hysteresis curves with slim width and fairly small amount of remanence magnetizations and coercive fields. As shown in the inset of Fig. 12 magnetization gradually increases with field. In addition to this, the M_r_/M_s_ ratio of a hysteresis curve is ranging from 0.085 to 0.109 which are quite small, thereby, the curves look like S form not square like. Hence, such a hysteresis loop could hardly be considered as superparamagnetic behaviour.

**Figure 13:**
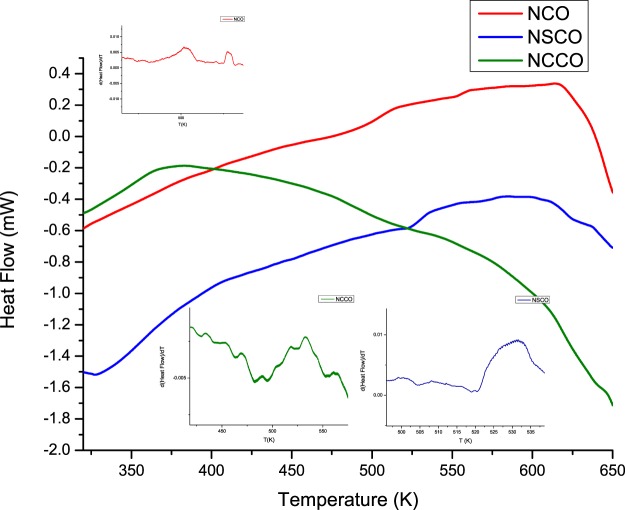
Thermal analysis of the samples by means of DSC. Inset figures shows the first derivative of heat flow with respect to temperature which indicates the related Curie Temperatures (T_C_).

Although Zero Field Cooling (ZFC) and Field Cooling (FC) Magnetization versus Temperature (M-T), of course, give additional information on magnetic behavior, above discussion on the basis of the room temperature M-H measurements and summarized parameters in Table 3 are enough to judge the magnetic behavior of the samples. As a matter of fact, ZFC and FC Magnetization versus Temperature (M-T), of course, give additional information on magnetic behavior for instance related Curie temperature (T_C_), thermal analysis like Differential Scanning Calorimetry (DSC) is able to determine T_C_ of a magnetic material. So, the DSC measurements carried out for all the samples indicated that Curie temperatures (T_C_) were all highly above room temperature as seen in Fig. 13. Therefore, T_C_ of NCO, NSCO, and NCCO were precisely determined from DSC measurements according to the endothermic peaks and inset figures which show the first derivative of the heat flow with respect to temperature by the following values of 462, 520, and 495 K, respectively.

**Figure 14:**
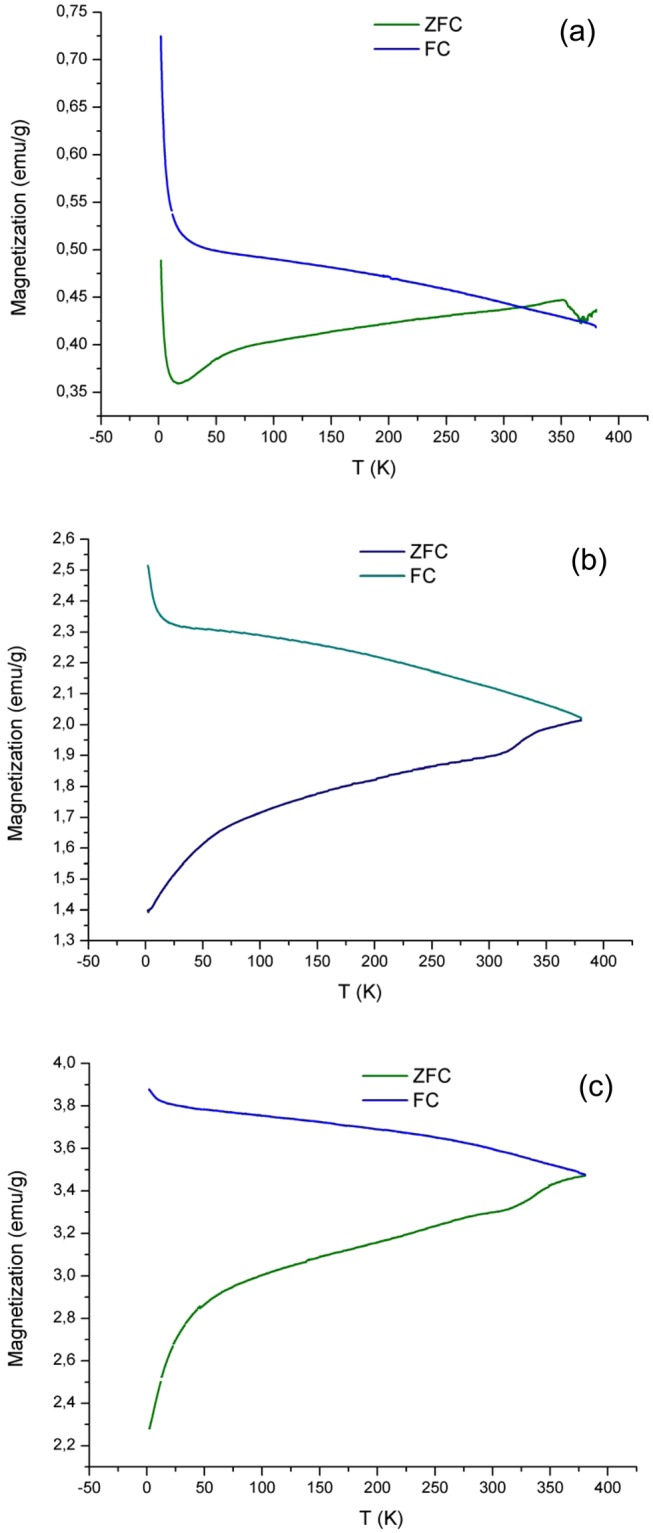
Temperature dependent magnetization (M-T) curves of (**a**) NCO, (**b**) NSCO, and (**c**) NCCO.

Temperature dependent magnetization (M-T) were performed by means of Zero Field Cooling (ZFC) and Field Cooling (FC) measurements between the limited temperature ranges of 2–380 K at 100 Oe. Corresponding M-T curves are presented in Fig. 14. Common behavior of the ZFC and FC curves is that the ZFC magnetization decreases continuously by decreasing temperature while that of FC increase. The ZFC curve of NCO presents a peak at around 350 K which is interpreted as the Blocking temperature (T_B_). Below T_B_ ZFC and FC curves significantly split. Regarding NSCO and NCCO, due to the limitation of temperature range we were not able to present the behavior of ZFC and FC curves above 380 K. On the other hand, since the splitting temperatures for NSCO and NCCO are higher compared to NCO it seems that Blocking temperatures and Curie temperatures are higher than those of NCO.

Possible formation of vacancies and defects near surface region and grain boundaries in crystal structure are important in stabilizing ferromagnetism due to the discontinuity of magnetic moment configuration in the system. Therefore, while some of imperfections can assist to ferromagnetism some of them may disturb it in this context^50^. Because the size of nanoparticles is small the fraction of the surface atoms is high in nanoparticle systems^51^. Hence, remarkable contribution to magnetization arises from the surface. However, different types of defects and imperfections such as lattice disorders, dangling bonds, and vacancies are present at the surface that causes to unbalanced broken spins^52,53^. This is the origin of the surface magnetization which is a generic property of nanoparticles^54^.

Mechanical milling causes for structural disorders in grains and their boundaries. Due to gradually increasing temperature some of defects inside the grains are able to move to grain boundaries^55^. Hence, the degrees of disorders near the surface region will enhance and lead to pin mobile charges. Due to the localization of charged particle resistivity will be increased^56^ as evidenced by decrease of dielectric loss in this context, which leads to enhance the soft ferromagnetic behaviour in some sense.

According to above discussion magnetization vs. magnetic field (M-H) hysteresis curve demonstrates that all the samples are soft ferromagnetic at room temperature. Low coercive fields are attributed to that magnetic domain walls can easily migrate, so the domains are reversed by low applied magnetic field. Additionally, they are well saturated even at low field. Moreover, there is small energy loss during magnetic cycling. Hence, such kinds of materials having slim M-H hysteresis loops are called as soft ferromagnetics. Substitution of Sr or Ca for Nd site seems to improve magnetic properties. Compared to parent NdCoO_3_ (NCO) both double remanence magnetization (2M_r_) and double coercivity (2H_C_) are enhanced. 2M_r_-2H_C_ values of NCO, NSCO, and NCCO are 4.22–348, 4.45–477, and 4.40 emu/g-361 Gauss, respectively. Therefore, since these soft ferromagnets require low energy to realign the magnetic domains for switching they may have potential applications for recording heads, magnetic cores, and transformers.

**Figure 15:**
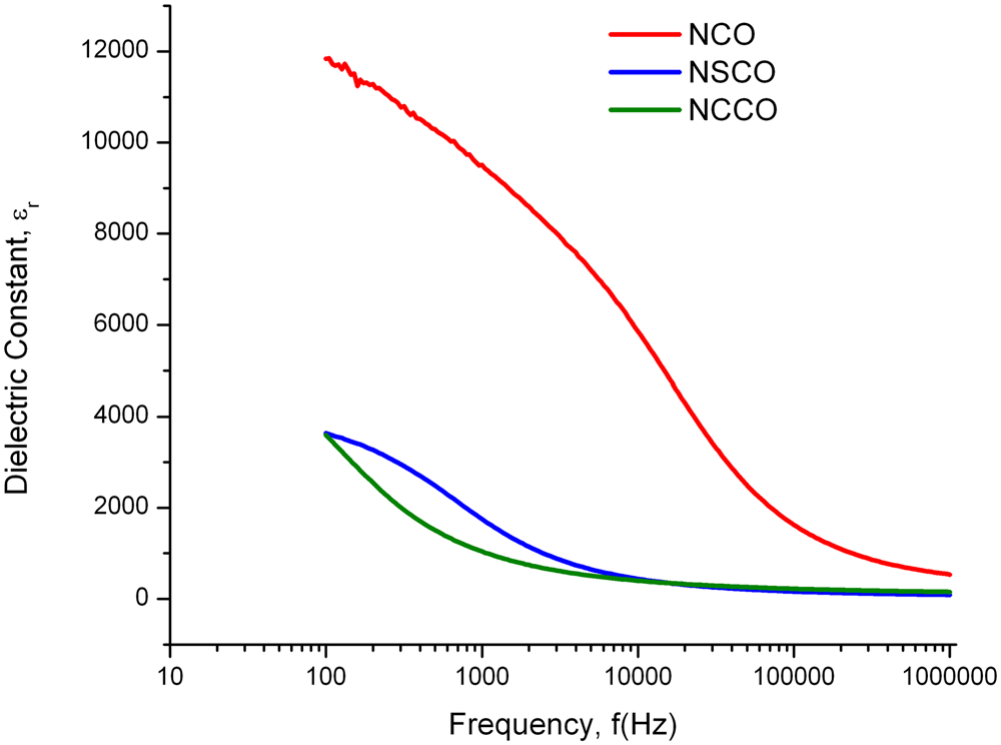
Variation of relative dielectric constant (ε_r_) of NCO, NSCO, and NCCO by frequency.

Dielectric relaxation of various perovskite type materials arises from conduction or dipolar origins^57^. Room temperature dielectric properties were carried out in the range of 100 Hz–1 MHz. Figure 15 shows relative dielectric constants which decreases with increasing frequency. Low frequency dielectric behavior is attributed to the space charge polarization, interfacial polarization and mobile charges. Since such polarization and mobile charges are not able to follow the electric signal at high frequencies, the dielectric constant decreases and stabilizes beyond 10 kHz for modified samples.

**Figure 16:**
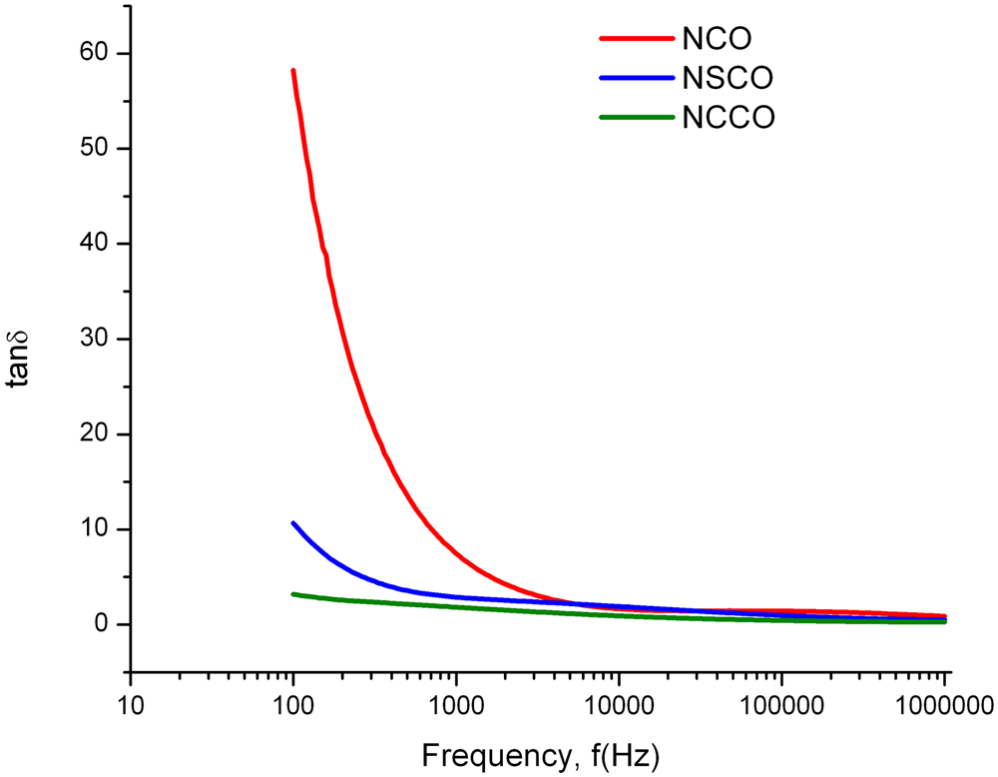
Variation of dielectric loss tangent of NCO, NSCO, and NCCO by frequency.

Figure 16 presents the dielectric loss. Loss tangent values decrease by increasing frequency by the same sense of dielectric constant. Relatively high conducting behavior at low frequencies is associated with polarization charges and charge exchange between Co ions. Samples having less loss have low dielectric constant. Therefore, higher resistivity of these soft ferromagnetic perovskite oxides will probably utilize them in high frequency device applications.

## Conclusions

In the present study, the NdCoO_3_ (NCO), Nd_0.8_Sr_0.2_CoO_3_ (NSCO) and Nd_0.9_Ca_0.1_CoO_3_ (NCCO) perovskites were manufactured from nitrate salts ball milled for various periods of times from 4 h to 48 h and they were subjected to heat treatment at 1000 °C for 2 h after 48 h milling. After all processes and analyses for NCO, NSCO and NCCO powders, for the first time, these materials were synthesised at nanocrystalline form by mechanical alloying technique which enable in only two steps, milling and heating. The following more conclusions are drawn:

1. NCO, NSCO and NCCO perovskites were successfully synthesized by mechanical alloying technique without any additional processes like other techniques.
2. The homogeneity of the NCO perovskite progressively continued with increasing milling time up to 48 h. Then, it was observed that each constituent was uniformly dispersed and the initial powders transformed into spherical form from irregular shapes.
3. The sharp crystalline peaks of XRD disappeared with increasing milling time and the perovskite peaks were obtained after heating at 1000 °C for 2 h for 48 h milling.
4. The crystallite sizes of the NCO, NSCO and NCCO perovskites decreased during mechanical milling. Thus, they were determined 18.2 nm, 19.2 nm and 19.7 nm, respectively, after 48 h of milling time. These values were confirmed by TEM.
5. All the materials exhibited soft ferromagnetic properties at room temperature.
6. The dielectric constants of the NCO, NSCO and NCCO perovskites decreased with increasing frequency.
